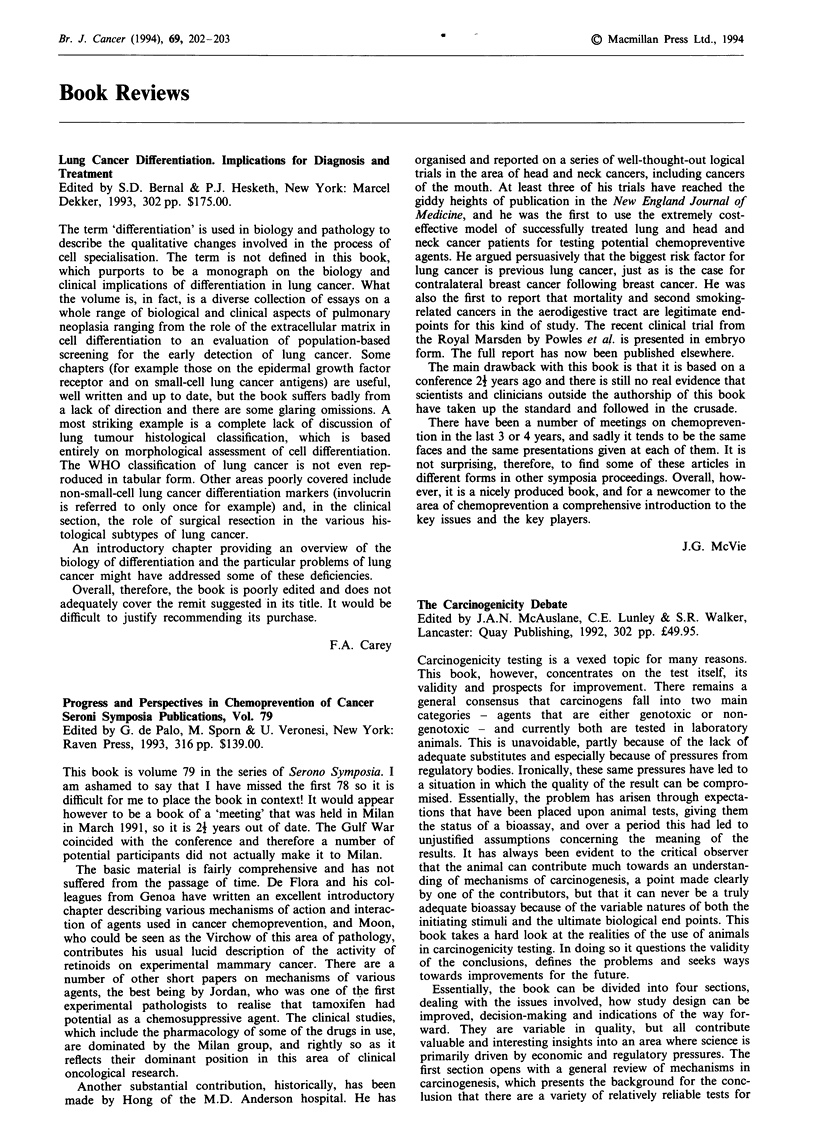# Lung cancer differentiation. Implications for diagnosis and treatment

**Published:** 1994-01

**Authors:** F.A. Carey


Br. J. Cancer (1994), 69, 202-203 © Macmillan Press Ltd., 1994

### Book Reviews

**Lung Cancer Differentiation. Implications for Diagnosis and Treatment**
Edited by S.D. Bernal & P.J. Hesketh, New York: Marcel Dekker, 1993, 302 pp. $175.00.

The term 'differentiation' is used in biology and pathology to describe the qualitative changes involved in the process of cell specialisation. The term is not defined in this book, which purports to be a monograph on the biology and clinical implications of differentiation in lung cancer. What the volume is, in fact, is a diverse collection of essays on a whole range of biological and clinical aspects of pulmonary neoplasia ranging from the role of the extracellular matrix in cell differentiation to an evaluation of population-based screening for the early detection of lung cancer. Some chapters (for example those on the epidermal growth factor receptor and on small-cell lung cancer antigens) are useful, well written and up to date, but the book suffers badly from a lack of direction and there are some glaring omissions. A most striking example is a complete lack of discussion of lung tumour histological classification, which is based entirely on morphological assessment of cell differentiation. The WHO classification of lung cancer is not even reproduced in tabular form. Other areas poorly covered include non-small-cell lung cancer differentiation markers (involucrin is referred to only once for example) and, in the clinical section, the role of surgical resection in the various histological subtypes of lung cancer.

An introductory chapter providing an overview of the biology of differentiation and the particular problems of lung cancer might have addressed some of these deficiencies.

Overall, therefore, the book is poorly edited and does not adequately cover the remit suggested in its title. It would be difficult to justify recommending its purchase.

F.A. Carey